# A Geriatric Assessment Intervention to Reduce Treatment Toxicity Among Older Adults With Advanced Lung Cancer: A Subgroup Analysis From a Cluster Randomized Controlled Trial

**DOI:** 10.3389/fonc.2022.835582

**Published:** 2022-03-31

**Authors:** Carolyn J. Presley, Mostafa R. Mohamed, Eva Culakova, Marie Flannery, Pooja H. Vibhakar, Rebecca Hoyd, Arya Amini, Noam VanderWalde, Melisa L. Wong, Yukari Tsubata, Daniel J. Spakowicz, Supriya G. Mohile

**Affiliations:** ^1^ Division of Medical Oncology, Department of Internal Medicine, The Ohio State University, Columbus, OH, United States; ^2^ Department of Medicine, University of Rochester Medical Center, Rochester, NY, United States; ^3^ Department of Surgery, University of Rochester Cancer Center National Cancer Institute (NCI) Community Oncology Research Program (NCORP) Research Base, Rochester, NY, United States; ^4^ Department of Radiation Oncology, School of Nursing, University of Rochester, Rochester, NY, United States; ^5^ Department of Radiation Oncology, City of Hope Comprehensive Cancer Center, Duarte, CA, United States; ^6^ West Cancer Center & Research Institute, Memphis, TN, United States; ^7^ Divisions of Hematology/Oncology and Geriatrics, Department of Medicine, University of California, San Francisco, San Francisco, CA, United States; ^8^ Division of Medical Oncology and Respiratory Medicine, Department of Internal Medicine, Shimane University Faculty of Medicine, Izumo, Japan

**Keywords:** treatment toxicities, geriatric assessment, lung cancer, older adult, clinical trial

## Abstract

**Introduction:**

More older adults die from lung cancer worldwide than breast, prostate, and colorectal cancers combined. Current lung cancer treatments may prolong life, but can also cause considerable treatment-related toxicity.

**Objective:**

This study is a secondary analysis of a cluster-randomized clinical trial which evaluated whether providing a geriatric assessment (GA) summary and GA-guided management recommendations can improve grade 3-5 toxicity among older adults with advanced lung cancer.

**Methods:**

We analyzed participants aged ≥70 years(y) with stage III & IV (advanced) lung cancer and ≥1 GA domain impairment starting a new cancer treatment with high-risk of toxicity within the National Cancer Institute’s Community Oncology Research Program. Community practices were randomized to the intervention arm (oncologists received GA summary & recommendations) versus usual care (UC: no summary or recommendations given). The primary outcome was grade 3-5 toxicity through 3 months post-treatment initiation. Secondary outcomes included 6-month (mo) and 1-year overall survival (OS), treatment modifications, and unplanned hospitalizations. Outcomes were analyzed using generalized linear mixed and Cox proportional hazards models with practice site as a random effect. ***Trial Registration:*** NCT02054741.

**Results & Conclusion:**

Among 180 participants with advanced lung cancer, the mean age was 76.3y (SD 5.1), 39.4% were female and 82.2% had stage IV disease. The proportion of patients who experienced grade 3-5 toxicity was significantly lower in the intervention arm vs UC (53.1% vs 71.6%, P=0.01). More participants in the intervention arm received lower intensity treatment at cycle 1 (56.3% vs 35.3%; P<0.01). Even with a cycle 1 dose reduction, OS at 6mo and 1 year was not significantly different (adjusted hazard ratio [HR] intervention vs. UC: 6mo HR=0.90, 95% CI: 0.52-1.57, P=0.72; 1 year HR=0.89, 95% CI: 0.58-1.36, P=0.57). Frequent toxicity checks, providing education and counseling materials, and initiating direct communication with the patient’s primary care physician were among the most common GA-guided management recommendations. Providing a GA summary and management recommendations can significantly improve tolerability of cancer treatment among older adults with advanced lung cancer.

## Introduction

Over 75% of all new non-small cell lung cancer (NSCLC) diagnoses are among adults ≥65 years of age (1). As lung cancer is generally a disease of older adults, cancer and aging research is significant because the population of older adults is large and growing. By 2030, nearly two-thirds of all cancer diagnoses will be among older adults (2, 3). More older adults die from lung cancer worldwide than any other cancer type (1). In the United States, among adults ≥65 years, 289 men and women per 100,000 will develop lung cancer (4). However, clinical trials include almost exclusively younger adults, thereby limiting external generalizability of clinical results to older adults, particularly in regard to toxicity and response to novel cancer drugs (5, 6). With the rapid approval of novel cancer drugs, the lack of evidence among older adults in pivotal trials continues to grow (7). A lack of clinical trial evidence perpetuates uncertainty for clinicians, patients, and families regarding important clinical outcomes such as treatment-related toxicity among older adults receiving lung cancer treatment within the community oncology setting. In addition, clinical trials include the healthiest older adults with little to no information on older adults with complex geriatric conditions.

Prior research has demonstrated that older adults with cancer have a high prevalence of characteristics that are associated with a greater risk of chemotherapy toxicity (8, 9). A geriatric assessment (GA) can identify areas of vulnerability (e.g., functional impairment, cognitive impairment, polypharmacy) and thus direct GA-guided management for older adults receiving cancer treatment (10–13). The GA has great potential to identify areas of vulnerability and develop recommendations that could help improve outcomes (e.g., treatment toxicity) among older adults with cancer (14–16). However, this type of evaluation is not routinely incorporated into the oncology clinical evaluation. A critical knowledge gap exists in respect to whether provision of GA information along with GA-guided management recommendations to the oncology treatment team would improve outcomes among older adults with advanced lung cancer receiving cancer treatment with a high risk of toxicity.

Balancing the benefits and risks of chemotherapy in the older adult patient population with advanced cancer is challenging because of the dearth of evidence-based data to guide these decisions (17, 18). Furthermore, older patients who are treated with chemotherapy are at high risk for adverse outcomes, including chemotherapy toxicity and functional and physical consequences (19–21). In addition, older adults are more susceptible to toxicity from combination chemotherapy plus newer immunotherapy or targeted kinase inhibitors (22–25). In a randomized controlled trial by Corre et al, GA-guided lung cancer treatment strategies have been shown to lower symptomatic toxicities and improve other clinical outcomes among older adults receiving chemotherapy for advanced lung cancer (26). There was no difference seen in overall survival between the GA-directed arm versus usual care; yet, 23% of the patients treated in the GA-directed arm did not receive chemotherapy. A more recent large cluster-randomized controlled trial (GAP-70+) demonstrated that GA-guided management recommendations could decrease the proportion of older adults who experienced a serious grade 3-5 toxicity from a new cancer treatment regimen for advanced cancer (>80% had stage IV disease) (27). A lower proportion of patients in the intervention arm experienced grade 3-5 toxicity (177/349; 50.7%) than in usual care (263/369; 71.3%); relative risk (RR) was 0.74 (95% CI: 0.64-0.86; p<0.001) (27). GA-guided recommendations can focus on managing symptomatic toxicities from cancer treatment among patients with functional impairments or can be interventions that are known to improve outcomes of older adults with geriatric syndromes (e.g., physical therapy and fall prevention education in patients who are falling or who are at risk for falling).

The primary goal of this secondary GAP-70+ analysis was to evaluate whether providing a GA summary and GA-guided management recommendations could decrease grade 3-5 toxicity specifically among older adults with advanced lung cancer.

## Methods

### Study Design

This is a secondary data analysis of the participants with lung cancer from the cluster-randomized clinical trial entitled “Geriatric Assessment for Patients 70 years and older (GAP-70+; NCT02054741).” Community oncology practices within the National Cancer Institute Community Oncology Research Program (NCORP) were randomized to the intervention arm (oncologists received GA summary & management recommendations) or usual care (UC: no summary or recommendations given; notifications were provided to oncologists for patients who screened positive for depression and severe cognitive impairment). NCORP practices were recruited through the University of Rochester National Cancer Institute (NCI) Research Base network (UR NCORP). NCORP is a national network of community cancer clinical trial practice sites in the United States (https://ncorp.cancer.gov/about/). Practice clusters were comprised of NCORP-affiliated community oncology practices. Participating practice clusters represent a large geographic area across the United States of which 33/40 practices enrolled patients with lung cancer. The UR Research Base coordinated study activities, but the UR did not enroll participants. The UR (Rochester, NY, USA) and all participating practice clusters obtained approval from their institutional review boards. All patients completed informed consent.

### Participants

Participants were recruited from July 2014-March 2019. Participants aged ≥70 years(y) with advance solid tumors or lymphoma and ≥1 GA domain impairment (other than polypharmacy) starting a new cancer treatment regimen with a high risk of toxicity within 4 weeks of enrollment were included. Participants were required to be able to understand English and provide written informed consent independently or with a healthcare proxy. For inclusion in this secondary analysis, participants with advanced (non-surgical stage III/IV) lung cancer, either NSCLC or extensive stage small cell lung cancer (ES-SCLC), were selected. Treatment regimens had to include at least one chemotherapy agent or have a >50% prevalence of grade 3-5 toxicity as determined by the primary oncologist with review and approval by a clinical team blinded to study arm at the Research Base (27, 28). The treating oncologists selected the specific treatment regimen, dosing, and schedules.

### Procedures

Community oncology practice clusters were randomized to the GA intervention versus UC arm, stratified by large or small based on prior accrual records. Participants in both arms completed a GA and were asked about proposed treatment plan before starting a new treatment regimen. Participants in the intervention arm were additionally given recommendations before starting a new treatment regimen. Oncologists in the intervention arm were provided with a tailored GA summary and GA-guided management recommendations before any cancer treatment was initiated. The GA evaluated 8 domains: comorbidity, cognition, physical performance, functional status, nutritional status, social support, polypharmacy, and psychological health. The recommendations provided based on GA domain impairment can be found in detail in the supplemental documents of Mohile et al. (27) Oncologists in the UC arm received notification for depression or severe cognitive impairment on screening tests, but no management recommendations were provided. There was no patient or provider blinding as this study evaluated a model of care rather than a particular treatment agent; however, all research investigators were blinded to the site assignment when the treatment and toxicity data were reviewed centrally.

### Outcomes

The primary outcome was grade 3-5 toxicity within 3 months of starting a new treatment regimen. Secondary outcomes included unplanned hospitalizations, subsequent dose reduction, dose delay, treatment discontinuation, overall survival (OS) at 6-month (mo) and 1-year in addition to cycle 1 treatment intensity (standard vs reduced). Practice staff prospectively captured toxicities over 3 months using NCI’s Common Terminology Criteria for Adverse Events (V4.0). Blinded oncology clinicians reviewed medical records to verify all treatment and toxicity data. At UR NCORP, two blinded clinicians reviewed each enrolled patient’s medical record and treatment regimen and used guidelines and clinical trials to determine standard dosing and length for treatment regimens. We evaluated the proportion of patients who received a reduced intensity regimen (e.g., lower dose or omission of an agent compared to standard) at cycle one. Standard treatment was evaluated according to National Comprehensive Cancer Network guidelines (29) of published phase II/III clinical trials. The blinded clinicians also reviewed medical records to evaluate unplanned hospitalizations (an overnight hospital stay for any reason that was not scheduled), dose reductions, dose delays, and treatment discontinuation. These were assessed by comparing what the patient received compared to what was planned by the oncologist at the start of treatment. Outcomes captured those changes related to clinical reasons (e.g., toxicity, patient preference) but not logistical reasons (e.g., holiday).

### Statistical Analysis

Descriptive statistics were performed to summarize demographics, GA measures, baseline clinical characteristics, and outcome measures. Bivariate analyses using chi-square tests for categorical variables and *t* tests for continuous variables were done to compare differences between study arms. A Generalized Linear Mixed Model (GLMM) was applied to analyze the primary outcome of grade 3-5 toxicity within 3 months with practice site as a random effect and study arm as a fixed effect. Proportions of patients who experienced grade 3-5 toxicity in the intervention vs UC arm were calculated by odds ratio adjusted for practice site. Kaplan-Meier method was used to estimate 6-month and 1-year OS and the effect of the intervention on OS was assessed by Cox Shared Frailty Model with practice sites as random effects. Similar to the primary outcome, GLMMs were applied to evaluate secondary outcomes (hospitalization, subsequent dose reduction, dose delay, treatment discontinuation, and reduced treatment intensity at cycle 1). Two-sided p values of <0.05 were considered statistically significant. All analyses were conducted using SAS 9.4 (SAS Institute, Cary, NC).

## Results

Among 180 participants with advanced lung cancer (NSCLC + ES-SCLC), the mean age was 76.3y (range 70-91, SD 5.1), 39.4% were female and 82.2% had stage IV disease. Patients in both arms (64 participants in the intervention and 116 participants in the UC arm) had similar baseline characteristics including age, sex, race/ethnicity, marital status, education, and income (**Table 1**). The majority of participants received platinum doublet chemotherapy (>70%). The GA domain impairments had similar distributions across arms. The mean number of geriatric impairments was 4.7 (SD: 1.5) and did not differ between study arms. The physical performance domain impairment was the most prevalent GA impaired domain (>90% in both arms). This was followed by polypharmacy, comorbidity, functional status, and nutritional domain impairment (**Table 1**).

**Table 1:** Patient Characteristics by Study Arm.

	All patients	Intervention arm	Usual care arm	P-values
	(N = 180)	(N = 64)	(N = 116)	
**Age (mean [standard deviation])**	76.3 (5.1)	76.3 (5.3)	76.2 (4.9)	0.88
70-79	138 (76.7%)	46 (71.9%)	92 (79.3%)	0.12*
80-89	37 (20.6%)	17 (26.6%)	20 (17.2%)	
≥90	4 (2.2%)	0 (0.0%)	4 (3.5%)	
Missing	1 (0.6%)	1 (1.6%)	0 (0.0%)	
**Sex**				0.34
Male	108 (60.0%)	41 (64.1%)	67 (57.8%)	
Female	71 (39.4%)	22 (34.4%)	49 (42.2%)	
Missing	1 (0.6%)	1 (1.6%)	0 (0.0%)	
**Race/Ethnicity**				0.22*
Non-Hispanic White	164 (91.1%)	55(85.9%)	109 (94.0%)	
Black	7 (3.9%)	3 (4.7%)	4 (3.5%)	
Others	8 (4.4%)	5 (7.8%)	3 (2.6%)	
Missing	1 (0.6%)	1 (1.6%)	0 (0.0%)	
**Marital Status**				0.46*
Single, Never Married	3 (1.7%)	2 (3.1%)	1 (0.86%)	
Married/Domestic Partnership	111 (61.7%)	40 (62.5%)	71 (61.2%)	
Separated/Widowed/Divorced	65 (36.1%)	21 (32.8%)	44 (37.9%)	
Missing	1 (0.6%)	1 (1.6%)	0 (0.0%)	
**Education**				0.99
<High school	36 (20.0%)	13 (20.3%)	23 (19.8%)	
High school graduate	58 (32.2%)	20 (31.3%)	38 (32.8%)	
Some college or above	85 (47.2%)	30 (46.9%)	55 (47.4%)	
Missing	1 (0.6%)	1 (1.6%)	0 (0.0%)	
**Income**				0.49
≤$50,000	100 (55.6%)	39 (60.9%)	61 (52.6%)	
>$50,000	39 (21.7%)	12 (18.8%)	27 (23.3%)	
Decline to answer	40 (22.2%)	12 (18.8%)	28 (24.1%)	
Missing	1 (0.6%)	1 (1.6%)	0 (0.0%)	
**Cancer stage and lung cancer type**				0.05*
Stage III NSCLC	30 (16.7%)	16 (25.0%)	14 (12.1%)	
Stage IV NSCLC	148 (82.2%)	48 (75.0%)	100 (86.2%)	
ES-SCLC	2 (1.1%)	0 (0.0%)	2 (1.7%)	
**Prior chemotherapy**	28 (15.6%)	7 (10.9%)	21 (18.1%)	0.16
**Treatment Regimen (Chi-square test)**				
Chemo platinum doublet	134(74.4%)	45 (70.3%)	89 (76.7%)	0.38
Chemo+ immunotherapy	21 (11.7%)	8 (12.5%)	13 (11.2%)	
Single agent chemo	21 (11.7%)	8 (12.5%)	13 (11.2%)	
Other**	4 (2.2%)	3 (4.7%)	1 (0.8%)	
Number of Impaired Geriatric Assessment Domains** (mean [SD])	4.7 (1.5)	4.8 (1.5)	4.7 (1.4)	0.59
Physical performance domain impairment	167 (92.8%)	58 (90.6%)	109 (94.0%)	0.41
Polypharmacy domain impairment	151 (83.9%)	55 (85.9%)	96 (82.8%)	0.58
Comorbidity domain impairment	125 (69.4%)	45 (70.3%)	80 (69.0%)	0.85
Functional status domain impairment	115 (63.9%)	39 (60.9%)	76 (65.5%)	0.54
Nutrition domain impairment	124 (68.9%)	46 (71.9%)	78 (67.2%)	0.52
Cognition domain impairment	61 (33.9%)	23 (35.9%)	38 (32.8%)	0.67
Social support domain impairment	45 (25**·**0%)	20 (31.3%)	25 (21.6%)	0.15
Psychological status domain impairment	61 (33.9%)	21 (32.8%)	40 (34.5%)	0.82

*33% of the cells have expected counts less than 5. Chi-Square may not be a valid test.

**other included: targeted, targeted + chemo, or multiple chemo (no platinum).

**Figure 1:**
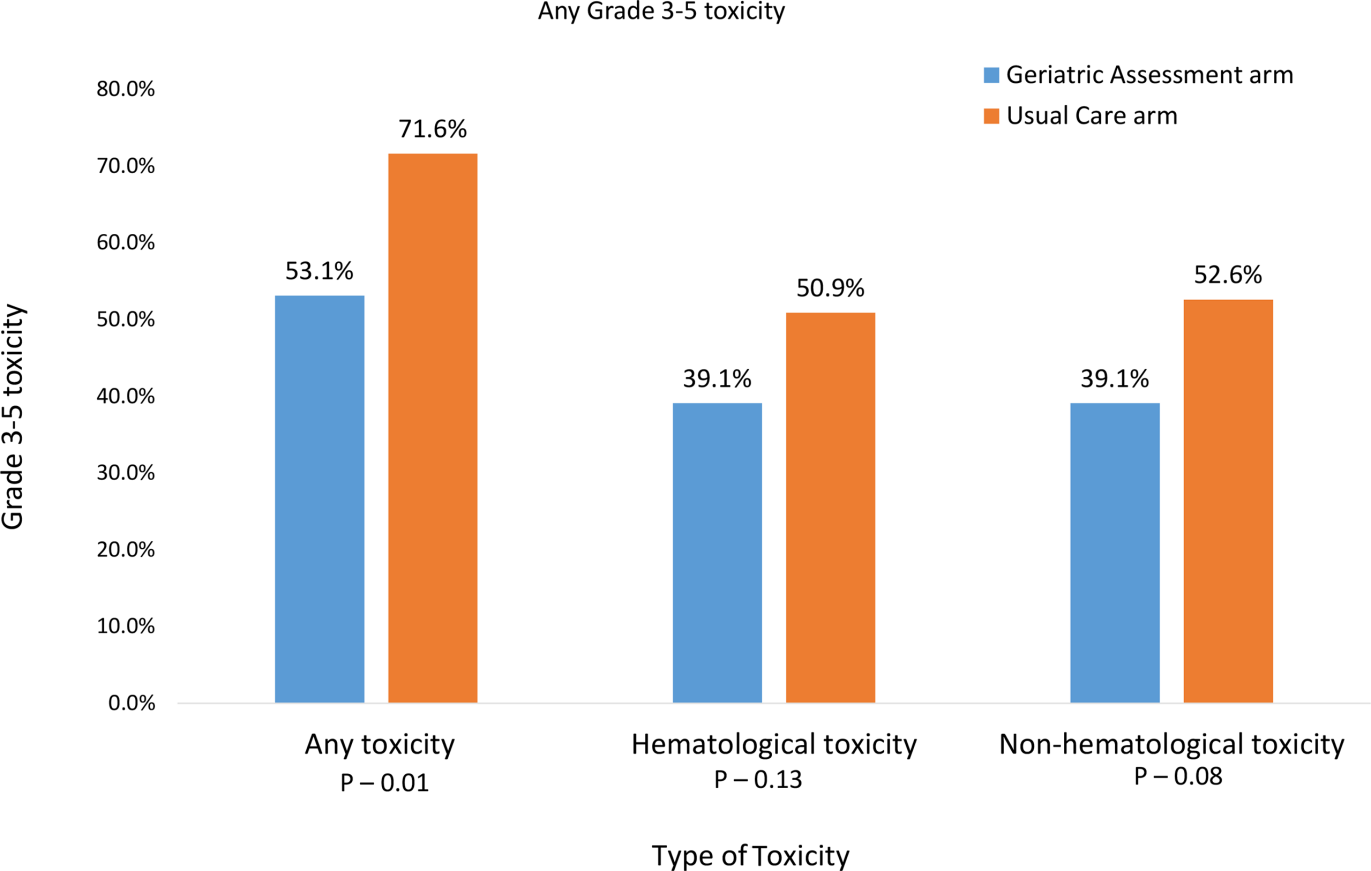
Prevalence of grade 3-5 toxicities over 3 months after the start of new treatment for advanced stage III/IV lung cancer.

**Figure 2:**
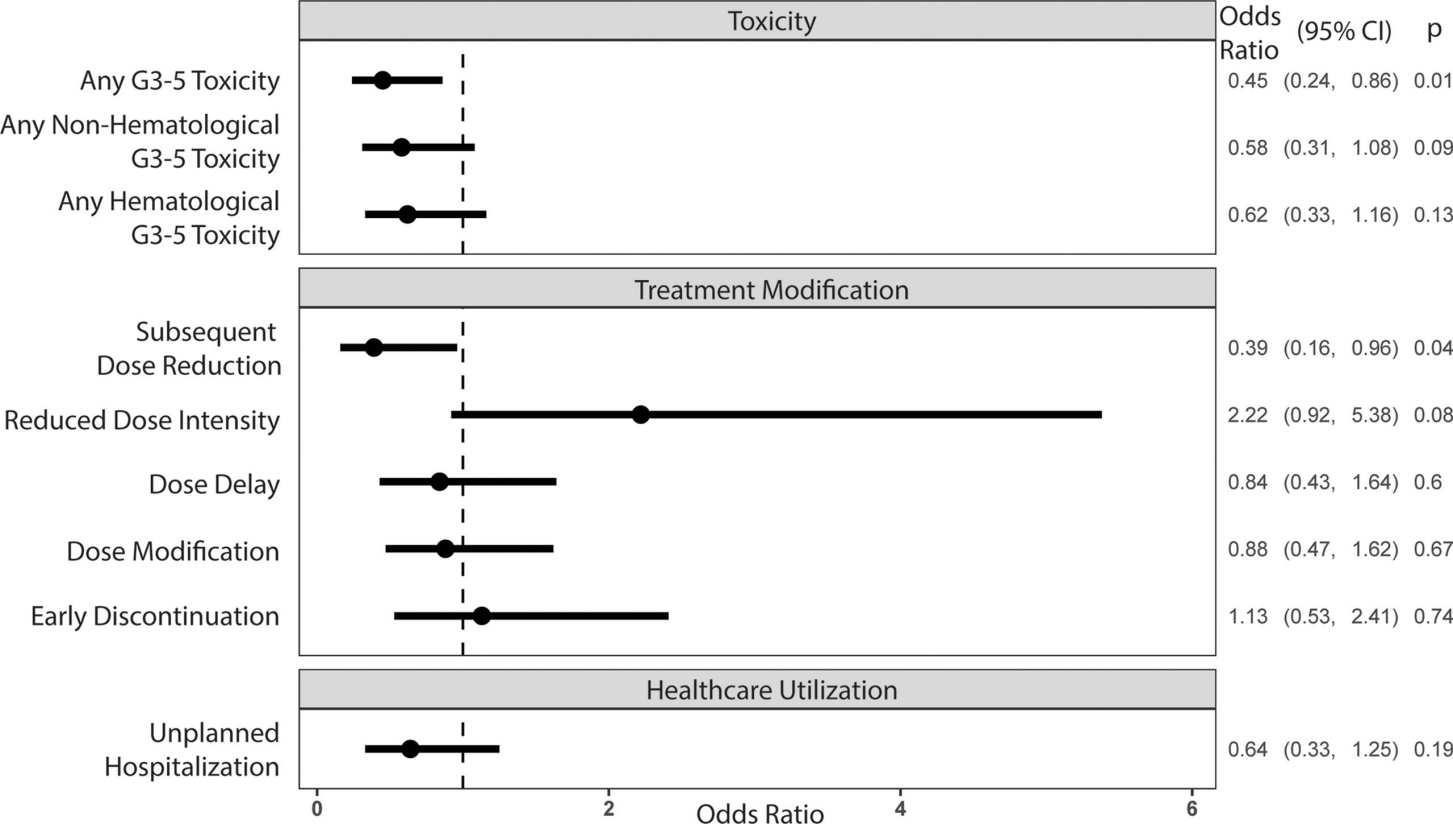
Odds ratios of outcome variables associated with the intervention arm, controlling for the site cluster (random effect)*. *All outcomes except reduced dose intensity at cycle 1 were assessed at 3 months of treatment.

Sixty-five percent of all participants experienced a grade 3-5 toxicity (**Figure 1**). The proportion of patients who experienced grade 3-5 toxicity was lower in the intervention vs. UC arm (53.1% vs 71.6%, P=0.01). After accounting for practice sites as a random effect, the odds of any grade 3-5 toxicity were lower in the intervention vs. UC arm (Adjusted odds ratio=0.45 95% CI: 0.24-0.86, P=0.01, **Figure 2**). More participants in the intervention group received lower intensity treatment at cycle 1 (56.3% vs 35.3%; P<0.01). Unplanned hospitalizations, dose delay, and early discontinuation were similar across groups. Subsequent dose reduction post-C1 was significantly higher in the UC arm (P=0.02, **Table 2**).

**Table 2:** GAP Study Lung Cancer Treatment Secondary Outcomes by Study Arm.

	All patients (n = 180)	GA arm (n = 64)	Usual care arm (n = 116)	P values
Unplanned Hospitalization	62 (34.4%)	18 (28.1%)	44 (37.9%)	0.19
Dose delay	55 (30.6%)	18 (28.1%)	37 (31.9%)	0.60
Subsequent dose reduction	40 (22.2%)	8 (12.5%)	32 (27.6%)	0.02
Early discontinuation of treatment	37 (20.6%)	14 (21.9%)	23 (19.8%)	0.74
Reduced dose intensity at cycle 1	77 (42.8%)	36 (56.3%)	41 (35.3%)	<0.01
Overall Survival at 6 months*	124 (68.9%)	45 (70.3%)	79 (68.1%)	0.76
Overall Survival at 1 year*	82 (45.6%)	31 (48.4%)	51 (44.0%)	0.56

*Censoring is not considered.

**Figure 3:**
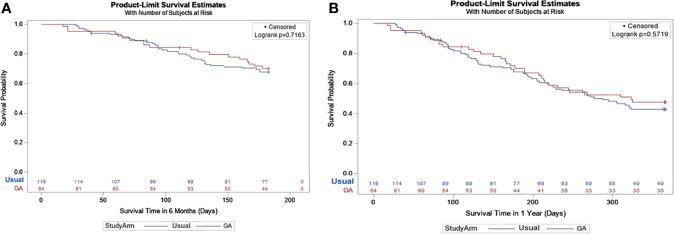
**(A)** Survival at 6 months based on Kaplan-Meier Estimates and Cox Model*. *Geriatric Assessment Intervention: 70.1% vs. Usual Care: 67.7%; Adjusted Hazard Ratio: 0.90 95% CI: (0.52-1.57), P = 0.72. **(B)** Survival at 1 year based on Kaplan-Meier Estimates and Cox Model*. *Geriatric Assessment Intervention: 47.8% vs. Usual Care: 43.1%; Adjusted Hazard Ratio: 0.89 95% CI: (0.58-1.36), P = 0.57.

The OS at 6mo and 1 year was not significantly different between arms (**Figures 3A** **, B**: adjusted hazard ratio [HR] interventions vs. UC: 6mo HR=0.90, 95% CI: 0.52-1.57, P=0.72; 1 year HR=0.89, 95% CI: 0.58-1.36, P=0.57). Frequent toxicity checks, providing education and counseling materials, and initiating direct communication with the patient’s primary care physician were among the most common GA-guided interventions recommended and acknowledged by the treating oncologist (**Table 3**).

**Table 3:** Geriatric assessment (GA) recommendations by domain.

Domains	Prevalence of the most common GA-guided management recommendations chosen by oncologists in the intervention arm
Comorbidity (n = 45 impaired in intervention arm)	- Initiate direct communication (written, electronic, or phone) with patient’s primary care physician about the plan for the patient’s cancer (90.0%) - Modify treatment choices if applicable to the individual patient. Examples: 1) History of diabetes - avoid neurotoxic agents if another option is equivalent (27.5%); 2) History of heart failure - minimize volume of agents and/or administer treatments at slower infusion rate (22.5%); 3) History of renal impairment-adjust as appropriate (25.0%) - Modify dosage or schedule if there is concern about how the patient will tolerate therapy or if there is a concern about worsening of comorbidities (42.5%) - Provide smoking cessation counseling if the patient currently smokes (7.5%)
Cognition (n = 23 impaired in intervention arm)	- Provide explicit and written instructions for appointments, medications, and treatment (77.3%) - Medication review - minimize psychoactive and high-risk medications (72.7%) - Assess decision-making capacity and elicit health care proxy information and input if the patient lacks decision- making capacity (45.5%) Cancer treatment decision – 1) modify dosage (e.g. 20% dose reduction with escalation as tolerated (40.9%); 2) modify treatment choice (consider starting with single agent with escalation to doublet if standard at second cycle depending on tolerance) (18.6%); 3) modify treatment regimen (e.g., use an option with demonstrated safety and efficacy in older and/or frail adults) (27.3%) - Give patient/family member handout on delirium risk counseling (22**•**9%) - Referral: refer to clinician experienced in memory care (9.1%) - Confirm someone else will help fill pillbox (54.5%)
Physical performance* (n = 58 impaired in intervention arm)	- Conduct frequent toxicity checks (89.7%) - Provide information on exercise and exercise prescription (87.2%) - Provide fall counselling hand-out/information (79.5%) - Provide hand-out on energy conservation (79.5%) - Medication Review: minimize psychoactive meds including those used for supportive care (28.2%); minimize duplicative medications (41.0%) - Treatment modification: consider modification of treatment dose or choice. Examples: 1) consider single agent rather than doublet therapy if appropriate (20.5%): 2) modify dosage (e.g., 20% dose reduction with escalation as tolerated) (51.3%); 3) modify treatment regimen (e.g., use an option with demonstrated safety and efficacy in older and/or frail adults) (46.2%) - Referrals: refer to 1) physical therapist (outpatient or home-based depending on eligibility for home care) (17.9%); 2) occupational therapist (7.7%); 3) aide services (7.7%); 4) personal emergency response information (25.6%); 5) vision specialist if difficulties (12.8%) - Physical Examination: check orthostatic blood pressure (23.1%) and decrease or eliminate blood pressure meds if blood pressure is low or low normal (12.8%)
Functional status* (n = 39 impaired in intervention arm)	
Nutritional status (n = 46 impaired in intervention arm)	- Conduct frequent toxicity checks (95.5%)- Give Nutrition hand-out (77.3%)- Give mucositis hand-out (72.7%)- Cancer Treatment: 1) use caution with highly emetogenic regimens and use another option if appropriate (81.8%); 2) utilize aggressive anti-emetic therapy (86.4%)- Referrals: refer to: 1) Nutritionist/Clinical Dietician (29.5%); 2) dentist if poor dentition or denture issues (2.3%); 3) speech and swallow if difficulty with swallowing (4.5%)
Social Support (n = 20 impaired in intervention arm)	- Confirm documented health care proxy is in medical record (77.8%) - Modify treatment choice and/or dosage (66.7%) - Provide referral or information on 1) Social worker *via* on-site or visiting nurse services (38.9%); 2) visiting nurse service or home health aide (if meets criteria) (16.7%); 3) transportation or ride services (22.2%); 4) medical insurance advising, advocacy, and negotiation (11**•**1%); 5) community resource mobilization (16.7%)
Polypharmacy (n = 55 impaired in intervention arm)	- Ask patient to bring in prescribed and over-the-counter medications and supplements to review at the next visit (45.3%) - Contact primary care provider to help reduce regimen complexity (17.0%) - Reduce medicines solely used for hypertension or diabetes if appropriate (including dose and number of medications) (17.0%) - Consult the pharmacist who fills the patient’s scripts to synchronize medication refills whenever possible (3.8%) - Have pharmacist meet with the patient to evaluate drug interactions and medication counseling (7.5%) - Recommend pillbox and/or medication calendar (30.2%) - Provide written instructions (at the sixth-grade level) to patient/caregiver for taking new medications (60.4%) - Provide hand out on polypharmacy (79.2%)
Psychological health (n = 21 impaired in intervention arm)	- Provide written or verbal communication with primary care physician (41**•**1%) - Referral: refer to 1) counseling or psychotherapy (9.5%); 2) social work (14.3%); 3) spiritual counseling or Chaplaincy services (14.3%); 4) palliative care if other physical and/or cancer symptoms are present (14.3%). - Initiate pharmacologic therapy if appropriate in conjunction with primary care provider (14.3%)

*Recommendations for physical performance and functional status impairments are combined and presented together.

ADL, Activity of Daily Living; OARS, Older American Resources and Services; TSH, thyroid stimulating hormone.

## Discussion

Providing GA information and recommendations can improve tolerability of cancer treatment among older adults with advanced lung cancer. Despite a significant difference in C1 dose reduction between arms (56.3% in the intervention arm versus 35.3% in the UC arm), there was no significant difference in 6-month or 1-year OS. However, there was a significantly decreased risk of grade 3-5 toxicity for the intervention arm. The majority of participants received a platinum-based chemotherapy regimen which is explained by the standard-of-care treatment at the time this study was conducted. The current standard-of-care is a platinum doublet with immunotherapy for most patients depending on PD-L1 status. Yet, our findings are still relevant to current treatment recommendations. With the addition of immunotherapy now to standard platinum doublets, the risk of toxicities is potentially even higher (30). Unfortunately, the proportion of older adults comprise only 41-55% of all patients with NSCLC included in the phase III clinical trials that led to the drug approvals (30), which are the healthiest of older adults. The incidence of high-grade toxicities among older adults with GA domain impairment receiving chemotherapy + immunotherapy is currently unknown.

This study confirms the utility of a GA among older adults with advanced lung cancer. The decrease in toxicity is similar to lung cancer outcome data presented by Corre et al. in the ESOGIA-GFPC-GECP 08-02 Study (26). Yet, a distinct difference is that GAP70+ is one of the first studies in the United States to provide geriatric domain-focused recommendations while letting the oncology team decide the final cancer treatment regimen. This is very distinct from the ESOGIA study that used the GA to dictate the lung cancer treatment regimen. The former approach is likely a much more palatable design for oncology clinicians in the United States, where personal and professional autonomy is culturally prioritized over algorithmic pathway approaches. This approach is also consistent with a current emphasis on shared patient-provider decision-making.

The majority of the GA was completed from patient-reported information. This may cause a barrier to implementation if the resources are not available either in-person or electronically to capture the patient-reported information. There are alternative GA tools (31, 32) such as the G8, the CARG, and CRASH tools that are shorter than the GA performed in this study; yet, many are not validated with the use of newer cancer therapeutics and do not include recommendations to the oncology team.

For advanced NSCLC in the United States, single agent immunotherapy (IO) is now a Food & Drug Administration-approved treatment option. Fewer patients who received single agent IO experienced grade 3-5 adverse events at 5 years of follow-up compared to those who received chemotherapy alone for PD-L1 positive (≥50%) disease (33). However, the trial comparing chemotherapy + IO versus IO alone (INSIGNA NCT NCT03793179) is ongoing. The PACIFIC study (34) also demonstrated an improvement in overall survival with the addition of durvalumab after concurrent chemoradiation. Unfortunately, over half of all older adults with advanced lung cancer are excluded from clinical trials (35). Future directions will hopefully explore GA-guided recommendations in a prospective clinical trial design among older adults with GA impairment receiving chemotherapy + IO for stage IV and chemoradiation therapy for stage III NSCLC. Whether to use concurrent versus sequential chemoradiation is controversial, but may have equivalent outcomes for older adults (36).

The majority of patients experienced impairment in physical performance and issues with polypharmacy. This is similar to the findings of Gomes et al. in a study of 70 older adults receiving IO for advanced NSCLC or malignant melanoma (37). Similarly, a study of over 200 older adults with lung cancer receiving treatment and GA demonstrated that handgrip strength was the most commonly impaired domain in octogenarians (38). Targeted interventions to improve both polypharmacy and physician impairment among other GA domain impairments are possible and should be incorporated into future research.

## Limitations

A very small number of older adults with ES-SCLC were included, which is not representative of the percentage of patients with SCLC in the United States. The majority received a platinum-based chemotherapy regimen, which was standard-of-care treatment at the time this study was conducted. This high number of platinum doublet may be higher than that of other countries and may not be necessarily generalizable to other countries or geographic regions. The standard of care treatment has also changed since the study period, and now includes a combination of chemotherapy + IO; in addition, older adults may have received single agent IO, which would not have met the high-toxicity regimen inclusion criteria. There was a higher number of stage IIIB patients in the intervention than the usual care arm, which could affect the secondary survival endpoints. Future studies would need to use survival as the primary endpoint and stage as a stratification factor for randomization. This study required a full GA assessment, which is often not possible in routine clinical cancer care. Due to the nature of this secondary data analysis and small sample size of the subgroup of patients with lung cancer, the analysis focusing on hematologic and non-hematologic toxicities separately and the secondary endpoints analyses may be lacking sufficient statistical power. Future prospective properly powered study may be needed to confirm these promising results. These limitations may reduce the overall generalizability of the study results.

## Conclusion

The use of a GA assessment and recommendations can result in upfront treatment dose reduction and a decrease in high-grade toxicity among older adults with advanced lung cancer without compromising survival outcomes. This is one of the first subset analyses in the United States to demonstrate the importance of GA recommendations in geriatric oncology treatment among older adults with advanced lung cancer.

## Data Availability Statement

The raw data supporting the conclusions of this article will be made available by the authors, without undue reservation.

## Ethics Statement

The studies involving human participants were reviewed and approved by The University of Rochester (Rochester, NY, USA) and all participating practice clusters obtained approval from their institutional review boards. The patients/participants provided their written informed consent to participate in this study.

## Author Contributions

SM and CP: conceptualization. SM, EC, MF, and MM: data collection. MM, EC, RH, and DS: data analysis. CP and PV: drafting original version. CP and SM: supervision. All authors read and approved the final version of this manuscript.

## Funding

This work was supported by the National Cancer Institute: R01CA177592, U01CA233167, UG1CA189961, The Ohio State University Comprehensive Cancer Center, and The National Institute of Aging (CP, 1K76AB074923-01, MW, K76AG064431, SM, K24AG056589, R33AG059206, DS, 1K01AG070310-01A1). Research reported in this publication was supported by The Ohio State University Comprehensive Cancer Center and the National Institutes of Health under grant number P30 CA016058.

## Conflict of Interest

MW receives royalties from UpToDate and immediate family member is an employee of Genentech with stock ownership. Dr. Flannery reports grants from NIH NCI RO1 CA 177592, grants from NIH NCI UO1 CA 233167, grants from NIH NCI UG1 CA189961, during the conduct of the study. YT received grant and personal fees from Daiichi Sankyo Co., Ltd and AstraZeneca K.K. and personal fees from Chugai Pharmaceuticals Inc. outside the submitted work.

The remaining authors declare that the research was conducted in the absence of any commercial or financial relationships that could be construed as a potential conflict of interest.

## Publisher’s Note

All claims expressed in this article are solely those of the authors and do not necessarily represent those of their affiliated organizations, or those of the publisher, the editors and the reviewers. Any product that may be evaluated in this article, or claim that may be made by its manufacturer, is not guaranteed or endorsed by the publisher.
